# Autophagy Stimulation Abrogates Herpes simplex Virus-1 Infection

**DOI:** 10.1038/srep09730

**Published:** 2015-04-09

**Authors:** Abraam M. Yakoub, Deepak Shukla

**Affiliations:** 1 Department of Microbiology and Immunology, University of Illinois, Chicago. IL USA, 60612; 2 Department of Ophthalmology and Visual Sciences, University of Illinois Medical Center, Chicago. IL USA, 60612

## Abstract

Herpes simplex virus-1 (HSV-1) is a double-stranded DNA virus that causes life-long infections. HSV-1 infections may lead to herpetic stromal keratitis that may advance to corneal blindness. HSV-1 infections can also cause fatal conditions, such as herpes encephalitis, or neonatal disease. A major virulence mechanism of HSV-1 is the control of autophagy, an innate immune defense strategy that could otherwise degrade viral particles. Here, to investigate a new mechanism for antiviral therapy, we tested the effect of various autophagy inducers (physiological and pharmacological) on infection. Autophagy stimulation was confirmed to significantly suppress HSV-1 infection in various cell types, without affecting cell viability. This study establishes the importance of autophagy for regulating HSV-1 infection, and provides a proof-of-principle evidence for a novel antiviral mechanism.

HSV-1 infects most humans worldwide, and causes significant healthcare concerns^1^. HSV-1 is the leading infectious cause of corneal blindness globally^2^, while central nervous system dissemination of the infection may result in fatal encephalitis^3^. Current HSV-1 therapy, mainly comprising nucleoside analogs such as acyclovir, suffers the significant drawback of emergence of resistant virus strains^4^ causing failure of treatment^1^,^4^, which emphasizes the need for investigating new mechanisms to control HSV-1 infections.

Macroautophagy (or, simply, autophagy) is a cellular process that degrades certain cytoplasmic components of the cell, or intracellular pathogens^5^. Autophagy involves sequestration of a part of the cytosol within isolation membranes, which then mature into double-membrane vesicles (autophagosomes) that eventually fuse with the lysosomes for lysosomal destruction of the cargo^6^. Autophagy plays an important role to combat bacterial or viral infections^5^,^6^,^7^. It was shown to limit the replication, or enhance the degradation, of various viruses^8^,^9^,^10^, in addition to its role in assisting processing and presentation of pathogen antigens, boosting the host adaptive immunity to infection^11^,^12^.

HSV-1 is a double-stranded DNA virus that controls host's autophagic responses through binding of the viral protein ICP34.5 to the host protein beclin1^13^, leading to inhibition of autophagy. Mutations of ICP34.5 lower virulence in mice^14^ and enhance viral degradation by autophagy^15^. Since control of autophagy is a robust virulence mechanism of the virus, we reasoned that enabling autophagy activation in infection may suppress the infection, and thus provide an unprecedented antiviral therapeutic tool. In this study, we investigate this novel concept.

## Results and Discussion

**Figure 1:**
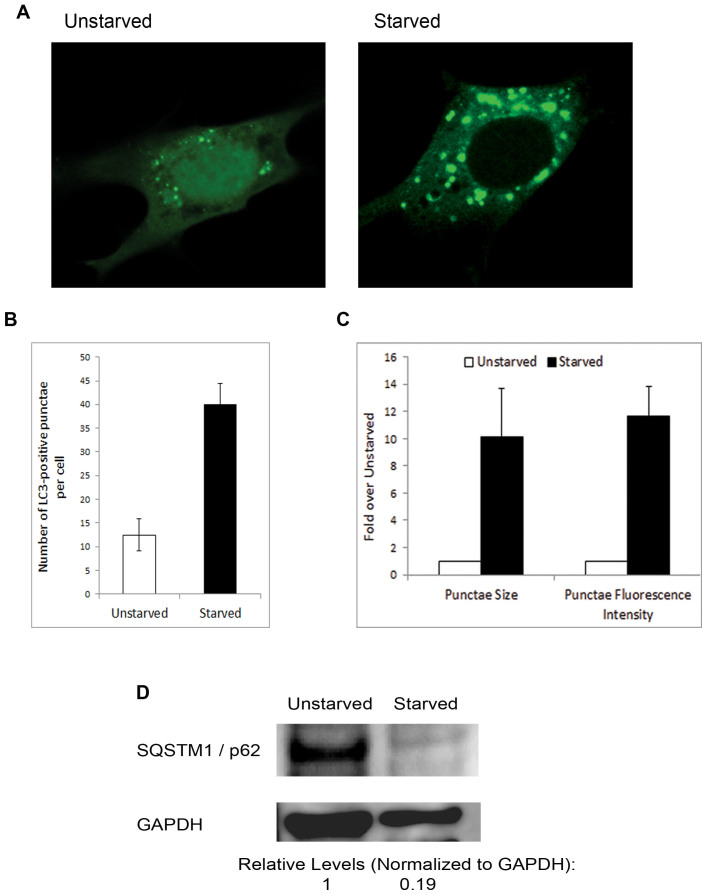
Validation of autophagy induction in cells. (A). MEFs were transfected with LC3-GFP. After 24 hrs, the cells were cultured in regular medium, or starved for 3 hrs. They were then fixed and processed for confocal microscopy imaging. (B). Quantification of the count of LC3-GFP punctae per cell; represents an average of 30 cells per sample. (C). Quantification of the area (size) and intensity of LC3-GFP punctae. Images were analyzed using MetaMorph software (Zeiss). An average of 30 cells was used for quantification. Shown is relative quantification (normalized to unstarved control; unstarved = 1). (D). Immunoblotting of SQSTM1/p62 from MEFs unstarved or starved for 16 hrs.

To investigate the effect of autophagy induction on HSV-1 infection, we induced autophagy in mouse embryonic fibroblasts (MEFs) via starvation. The cells were cultured in starvation medium for 3 hours, and then successful induction of autophagy was validated by multiple assays. Starved MEFs transiently expressing LC3-GFP (Ref. 16) were assessed for autophagy induction after starvation, using confocal microscopy. After treatment, the cells were fixed in paraformaldehyde, and imaged microscopically. While unstarved cells showed diffuse LC3 presence in the cell and only few LC3-GFP punctae (autophagosomes), starved cells showed enhanced autophagosomal development, as manifested by the increase in number, size and fluorescence intensity of LC3-GFP punctae which accumulated and clustered mostly in the cell cytoplasm (Figure 1A, B, and C). To further confirm persistent autophagy upregulation at later points in starved cells, we determined the levels of sequestosome1 (SQSTM1/p62), a protein degraded mainly by autophagy, using immunoblotting. Starved cells showed significantly decreased p62 levels, consistent with autophagy activation in the cells (Figure 1D).

**Figure 2:**
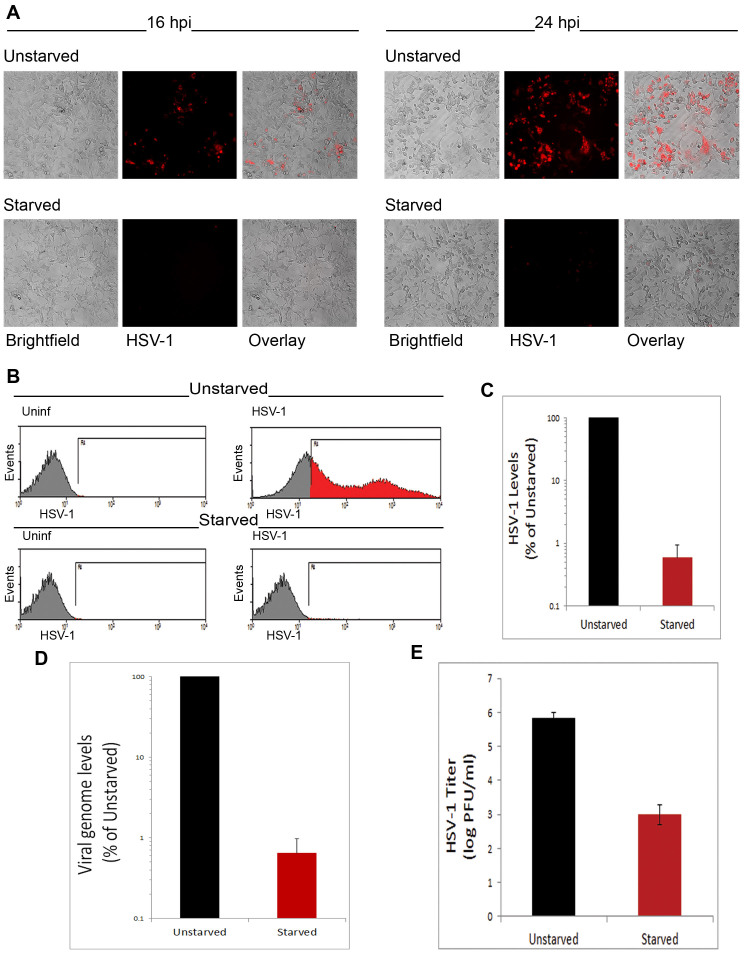
Suppression of HSV-1 infection under physiologically induced autophagy. (A). Unstarved or starved MEFs were infected with HSV-1-RFP (HSV-1) virus. The infection was monitored at 16 and 24 hours post-infection (hpi) using fluorescence microscopy. (B). FACS analysis of the cells in (A) at 24 hpi. (C). Quantification of HSV-1 levels, based on the percentage and MFI of the cell population in the positive region (R4) of the histograms in (B). (D). HSV-1 virion DNA was isolated from infected unstarved or starved MEFs at 24 hpi. Viral genomes were quantified using qPCR. (E). MEFs were infected with HSV-1 (incubation with the virus for 2 hr, then the virus was washed away). The cells were then replenished with regular or starvation medium. Supernatant was titered for virus levels ate 20 hpi.

Having validated autophagy induction by starvation, we then tested its influence on infection. Therefore, unstarved or starved MEFs were infected with a red fluorescent protein (RFP)-expressing HSV-1 virus. Then we monitored viral levels throughout the course of infection with fluorescence microscopy. We observed significant suppression of infection under starvation-induced autophagy (Figure 2A). FACS analysis of infected cells confirmed a significant block of HSV-1 infection upon autophagy induction (Figure 2B, and C). To further validate the effect of autophagy induction on viral levels, we isolated HSV-1 genomic DNA from infected cells, and quantified it using a quantitative polymerase chain reaction (qPCR) assay. HSV-1 genome quantification indicated that induced autophagy strongly suppresses HSV-1 infection (Figure 2D). Moreover, virus titer determination by plaque assay further confirmed this result (Figure 2E).

**Figure 3:**
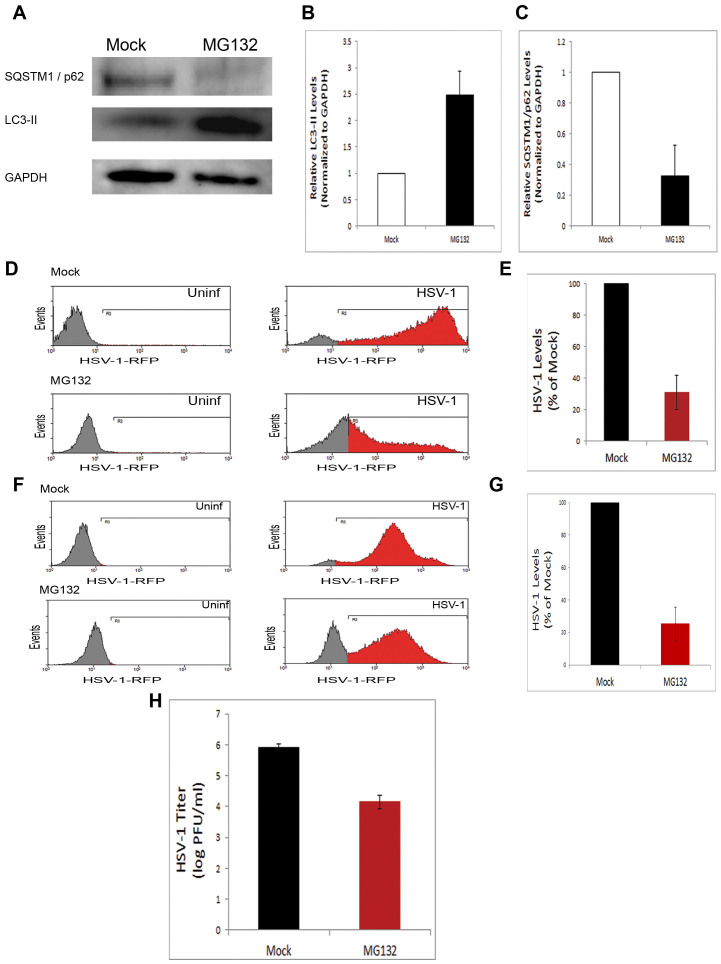
Suppression of HSV-1 infection under pharmacologically induced autophagy in various cell types. (A). Mock- or MG132-treated HCE cells were immunoblotted for SQSTM1/p62 and LC3. (B). Quantification of the relative LC3-II levels upon MG132 treatment in (A). (C). Quantification of the relative SQSTM1/p62 levels upon MG132 treatment in (A). (D). HCE cells were uninfected or infected with HSV-1-RFP (HSV-1) in the absence (Mock) or presence of MG132. The cells were analyzed cytofluorimetrically after 24 hr. (E). Quantification of (D), from multiple independent experiments, calculated using integrated MFI (percentage X MFI) of the RFP-positive population in the region R3. (F). Uninfected or HSV-1-RFP-infected RGC5 cells were mock-treated or treated with MG132, and analyzed cytofluorimetrically at 24 hpi. (G). Quantification of (F), calculated using integrated MFI of the RFP-positive population in the region R3. (H). MEFs were infected with HSV-1 for 20 hr, in presence or absence of MG132. Then the supernatant of infected cells was titered by plaque assay.

After confirming that induction of autophagy by starvation inhibits infection of cells, we then investigated whether this effect is cell-type dependent, especially considering that the role of autophagy in viral infection was once suggested to be cell type-specific^17^. Additionally, we also wanted to rule out the possibility that the infection-inhibiting effect of induced autophagy is specifically associated with a particular autophagy induction method. To address these questions, we employed another autophagy-inducing method (pharmacologically) and used various cell types infectable by HSV-1, and tested whether the effect of physiological autophagy induction on viral infection in MEFs may be recapitulated by other cell types such epithelial or neuronal cells. Thus, we treated human corneal epithelial (HCE) cells with the autophagy-inducing chemical benzyl (S)-4-methyl-1-((S)-4-methyl-1-((S)-4-methyl-1-oxopentan-2-ylamino)-1-oxopentan-2-ylamino)-1-oxopentan-2-ylcarbamate (known as MG132) at the concentration of 1 μM well known to induce autophagy^18^,^19^. Validation of autophagy stimulation was achieved via LC3-II and SQSTM1/p62 immunoblotting (Figure 3A, B and C). Moreover, a flow cytometry-based assay confirmed that MG132 treatment significantly upregulates autophagy levels (data not shown). To ensure equal viral entry and measure the effects of autophagy on viral replication, we added the autophagy-inducing agent after incubation of the cells with the virus (after viral entry). We then assessed viral levels in the cells by flow cytometry. We found that inducing autophagy in host cells led to a significant suppression of viral yields in host cells (Figure 3D, and E), as manifested by the significant drop in both the percentage and mean fluorescence intensity (MFI) of HSV-1-RFP-positive population in MG132-treated relative to mock-treated cells. Then we tested the influence of autophagy induction on viral infection in neuronal retinal ganglion (RGC) cells. We found that activating autophagy significantly hampers HSV yields and infection in these cells also (Figure 3F, and G). Virus titer determination by plaque assay also confirmed the effect of the autophagy-inducing agent on infection (Figure 3H). Taken together, these data along with starvation data demonstrate that autophagy induced via multiple means, physiologically (starvation) or pharmacologically, abrogates HSV-1 infections in a cell type-independent manner, suggesting that autophagy induction may be a new mechanism for antiviral drug development.

**Figure 4:**
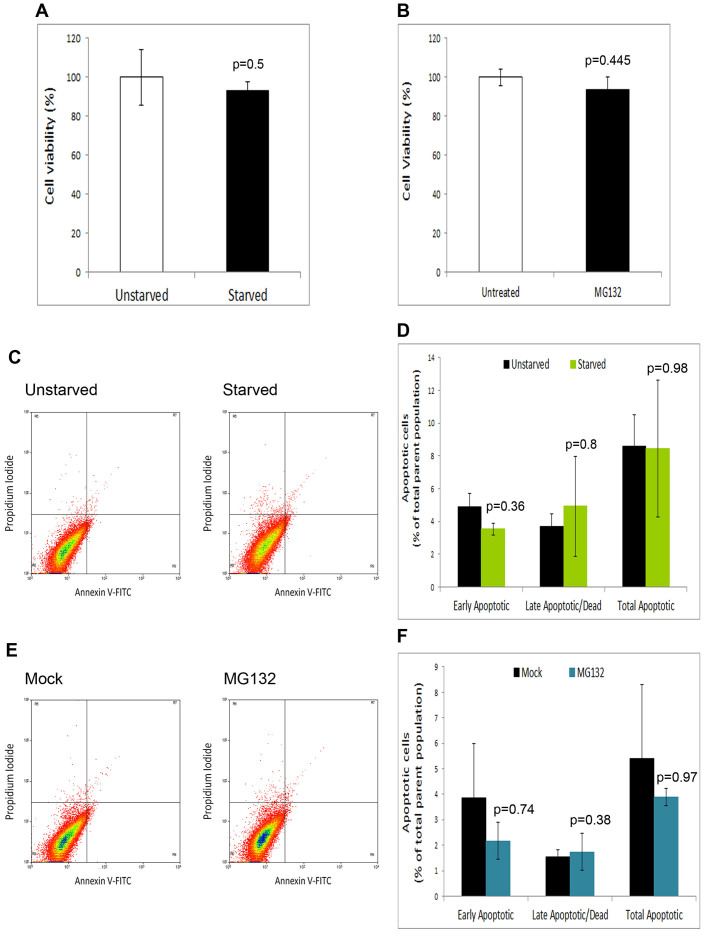
Effect of autophagy induction on cell viability. (A). MEFs were unstarved or starved for 20 hrs, and the cells were then assessed for viability using MTT cytotoxicity assay. (B). Cell viability (MTT assay) of mock- or MG132-treated HCE cells (24 hrs after treatment). (C). Annexin V/propidium iodide (PI) apoptosis assay of unstarved or starved cells. The flow cytometry histograms show early apoptotic cells (annexin V-positive/PI-negative population included in the quadrant R9), and late apoptotic or dead cells (annexin V-positive/PI-positive population included in the quadrant R7). (D). Quantification of the histograms in (C). (E). Annexin V/PI apoptosis assay of mock- or MG132-treated cells. (F). Quantification of the histograms in (E). Panels A, B, D, and F show p-values (t-test).

Having observed the high efficacy of autophagy stimulation on viral infection, to assess the possibility of pursuing autophagy induction for future drug development, we sought to test the effect of autophagy induction on cell viability. Activation of autophagy was shown to guard against protein synthesis halt, cell cycle delays, or cytotoxicity^20^,^21^,^22^,^23^,^24^,^25^. Indeed, autophagic activity of a cell was shown to be inversely proportional to the probability of its death^26^,^27^. Thus, we have closely monitored and validated by multiple means the activation of autophagy in treated cells, which should prevent cell death. To confirm that autophagy induction does not affect cell viability, starved or drug-treated cells were assessed for their health and viability, using microscopical observation of cells, and by measuring cell viability using a standard cytotoxicity assay (3-[4,5-dimethylthiazol-2-yl]-2,5 diphenyl tetrazolium bromide (MTT) cytotoxicity assay), as previously described^28^). We found that cell viability was not affected by either autophagy induction condition (Figure 4A, and B). To further confirm that autophagy upregulation does not influence cell viability or produce cell death, we performed annexin V-propidium iodide (PI) apoptosis/cell death FACS-based assay, a highly sensitive apoptosis assay that can also detect early apoptotic events^29^,^30^. We found that the autophagy induction methods used did not cause significant apoptosis or cell death (Figure 4C–F).

In this study, using information available on viral virulence mechanism and pathogenesis, we proposed and investigated a novel mechanism for viral killing (boosting a host defense mechanism, autophagy). Our results confirmed that inducing autophagic activity of host cells suppresses HSV-1 infection. These results give further evidence on the importance of autophagy for regulating HSV-1 infection, and suggest that autophagy induction may be a powerful means for suppressing viral infections by novel antiviral therapies. The strong efficacy (Figures 2 and 3), and the insignificant toxicity (Figure 4) associated with autophagy induction recommend such a mechanism as a highly efficient mechanism for new drug development. Importantly, autophagy regulates multiple viral infections, and many viruses tend to suppress or neutralize autophagy to achieve virulence^5^,^6^,^7^,^9^,^10^,^11^,^12^,^13^,^14^,^15^. In addition to HSV-1, other herpesviruses such as human cytomegalovirus (CMV) are known to suppress autophagy via multiple mechanisms^31^,^32^. Thus, it is possible to propose that autophagy stimulation may provide a broad-spectrum therapy against many viruses known to be regulated by autophagy. To conclude, we investigated a novel mechanism to control HSV-1 infections, and give evidence supporting the new concept of therapeutic utilization of autophagy, a currently emerging concept in medicine^33^,^34^.

## Methods

### Cells and cell culture

Human corneal epithelial (HCE) cells were from K. Hayashi (National Eye Institute, Bethesda, MD) and were cultured in Minimum Essential Media, MEM (Gibco) supplemented with antibiotics and 10% fetal bovine serum (FBS) (Sigma). Retinal ganglion cell line (RGC5) was provided by B. Yue (University of Illinois at Chicago), and was cultured in Dulbecco's modified Eagle's medium, DMEM (Gibco), supplemented with serum, antibiotics and amino acids. Mouse embryonic fibroblasts (MEFs) were a kind gift from C-A A. Hu (University of New Mexico, Albuquerque, NM). African green monkey foetal kidney epithelial (Vero) cells were from P. Spear (Northwestern University, Chicago, IL). MEFs and Vero cells were grown in DMEM (Gibco) supplemented with antibiotics and serum.

### Viruses

HSV-1 virus strain KOS was provided by P. Spear (Northwestern University, Chicago, IL). HSV-1(KOS)-RFP (HSV-1 KOS virus that expresses RFP conjugated to the capsid protein VP26) is a kind gift from P. Desai (The Johns Hopkins University, Baltimore, MD). Viruses were propagated and purified as previously described^28^.

### Plasmids

The plasmid pEX-GFP-hLC3WT (simply referred to as GFP-LC3) previously described^16^ was obtained from Addgene (plasmid number 24987).

### Antibodies

Polyclonal antibodies against LC3 were from Novus Biologicals (Catalog number NB100-2220). Polyclonal antibodies against GAPDH were purchased from Santa Cruz (Catalog number sc-25778). Polyclonal antibodies against SQSTM1/p62 were from Santa Cruz (Catalog number sc-25575). Horseradish peroxidase-conjugated secondary (anti-rabbit) antibodies were from Jackson Immunoresearch (Catalog number 111-005-144).

### Starvation

To induce autophagy by starvation, the cells were washed in Phosphate-buffered Saline (PBS) for three times, to remove residual medium. The cells were then cultured in Hanks' Balanced Salt Solution (Gibco).

### Infection

The cells were incubated with the virus in PBS containing 0.1% glucose and 1% serum at 37°C–5% CO_2_ conditions. After 2 hrs, the virus was removed and fresh medium was added to the cells (or Hanks' Balanced Salt Solution in case of starvation).

### Transfection

Transfections were performed using Lipofectamine2000 (Invitrogen) according to the manufacturer's protocols.

### Pharmacological induction of autophagy

For induction of autophagy, the proteasomal inhibitor benzyl (S)-4-methyl-1-((S)-4-methyl-1-((S)-4-methyl-1-oxopentan-2-ylamino)-1-oxopentan-2-ylamino)-1-oxopentan-2-ylcarbamate (known as MG132; Selleckchem., Catalog number S2619) was used at a concentration of 1 μM.

### Flow cytometry

After infection or treatment, the cells were washed in FACS buffer (PBS, 1% BSA), and analyzed cytofluorimetrically on LSRFortessa cytometer (BD). Data analysis was performed using Summit software (Beckman Coulter).

### Fluorescence microscopy

Following the infection, the cells were washed in PBS, and imaged using Axiovert 100 M fluorescence microscope (Zeiss). Image acquisition and analysis were carried out using MetaMorph software (Zeiss).

### Confocal fluorescence microscopy

For monitoring of LC3-GFP autophagosomal punctae, confocal microscopy (Zeiss 710 microscope, Zeiss) was used. After treatment or infection, the cells were washed, fixed in paraformaldehyde, and used in imaging. Image acquisition was performed using ZEN software (Zeiss), and image analysis was performed using MetaMorph software (Zeiss).

### HSV genome quantification

Infected cells were washed, and centrifuged. Cell pellets were suspended in buffer containing 1% SDS, 50 mM Tris (pH 7.5), and 10 mM EDTA, and the cell extract was incubated with proteinase K (50 μg/mL) at 37°C for 1 hr. DNA extraction then followed, via phenol/chloroform extraction-ethanol precipitation. Viral DNA was quantified using quantitative PCR (qPCR) on an ABI 7500 Fast thermocycler (Applied Biosystems), using HSV-specific primers. Primers used are: Forward (5′-TAC AAC CTG ACC ATC GCT TG-3′), and Reverse (5′-GCC CCC AGA GAC TTG TTG TA-3′), which detect the HSV glycoprotein D (gD) gene.

### Immunoblotting

Immunoblotting of proteins was performed as previously described^28^. Briefly, cells were harvested, lysed in RIPA buffer (Sigma, Catalog number R0278) containing protease-phosphatase inhibitors, and the lysates were electrophoresed through denaturing 4–12% SDS-polyacrylamide gel (Novex). Proteins were transferred onto a PVDF membrane, followed by block of non-specific binding with 5% non-fat milk in Tris-buffered saline (TBS). Membrane was then incubated with primary antibody and then with HRP-conjugated secondary antibody, followed by incubation with Femto-Sensitivity ECL (Thermo). Chemiluminescence was detected with ImageQuant LAS4000 digital image system (GE).

### Virus titer (plaque formation) Assay

Confluent monolayers of Vero cells were infected with serially diluted supernatants in PBS containing 0.1% glucose and 1% heat-inactivated serum for 2 hrs. After incubation, the cells were washed and methylcellulose (Sigma)-containing DMEM was added onto the monolayer. The cells were incubated at 37 C-5% CO_2_ conditions for 72 hrs, then fixed with methanol for 10 min at room temperature and stained with crystal violet. 30 min later, crystal violet was removed, monolayers were allowed to dry, and plaques were counted. Plaque counts were used to calculate viral titers.

### Cytotoxicity and apoptosis assays

Cytotoxicity was assessed via MTT (3-[4,5-dimethylthiazol-2-yl]-2,5 diphenyl tetrazolium bromide) assay. MTT was purchased from Sigma and the assay was performed according to the manufacturer's protocols and as previously described^28^. Apoptosis was assayed using FITC Annexin V/Dead Cell Apoptosis Kit (Molecular Probes, Invitrogen). The assay was performed according to the manufacturer's guidelines. Flow cytometry for annexin V and PI levels was then performed as described above, and data analysis was performed using Summit software (Beckman Coulter). Apoptosis analysis was performed in accordance with standardized guidelines^29^,^30^, and as previously described^35^,^36^.

### Statistical analyses

Experiments were independently performed for at least three times. Quantification shown in figures represents mean values; error bars represent standard error of the mean. Statistical significance was determined via Student's t-test (minimum p-value for significance 0.05). Unless otherwise indicated, the data were statistically significant (p-values less than 0.05).
